# From Diagnosis to Resolution: A Case Study of Myxopapillary Ependymoma Survival

**DOI:** 10.7759/cureus.68490

**Published:** 2024-09-02

**Authors:** Mohammed Al-Banna, Mahmoud Abughazal, Mustafa Aljanabi, Mohamed Hassan, Moustafa Abouelkheir

**Affiliations:** 1 Emergency Medicine, United Lincolnshire Hospitals NHS Trust, Boston, GBR

**Keywords:** lower back pain (lbp), neuro-surgery, neurological emergencies, spinal cord tumor surgery, myxopapillary ependymoma

## Abstract

Myxopapillary ependymoma (MPE) is a rare, slow-growing tumor that commonly arises in the lumbosacral region of the spinal cord, within the filum terminale and cauda equina. The frequent presentation of MPE is back, sacral, or leg pain. The tumor's size, site, and extension usually influence these symptoms. MPE is usually evaluated using magnetic resonance imaging (MRI) because of its superior soft tissue contrast. The best treatment modality is total surgical resection, which improves the long-term survival rate, with follow-up imaging recommended to ensure total resolution. Here, we present the case of a 29-year-old male who presented with symptoms suggestive of severe neurological impairment. An MRI scan revealed an intradural lesion arising from the cauda equina with peripheral and intrathecal haemorrhage, consistent with MPE. He was managed with laminectomy and microsurgical resection of the tumor, which achieved total resection. Postoperative follow-up found gradual improvement in his symptoms, and routine surveillance imaging confirmed the complete resolution of the tumor.

## Introduction

In 2021, the World Health Organization (WHO) classified myxopapillary ependymoma (MPE), a central nervous system ependymal tumor, as a grade 2 tumor [1]. Grade 2 ependymomas are still classified as low-grade tumors; however, they tend to exhibit a higher proliferative index and potentially more aggressive behavior than grade 1 ependymomas, especially in cases where complete surgical resection is not achieved [2]. MPEs represent less than 1-5% of all spinal cord neoplasms and 13% of all spinal ependymomas. MPE typically affects adults between the ages of 30 and 50 [3]. Despite its generally benign histology, MPE can lead to significant morbidity due to its location and potential for local invasion and cerebrospinal fluid (CSF) dissemination [3]. There is no specific risk factor for MPE. However, compression of the spinal nerve roots by the tumor can lead to characteristic symptoms, including radicular back and extremity pain, bladder and bowel dysfunction, lower extremity weakness, sensory loss, and paraesthesia, particularly when the lumbar spine is affected [4].

Magnetic resonance imaging (MRI) is the diagnostic modality of choice for spinal cord tumors, providing superior soft tissue contrast and detailed visualization of the tumor’s extent and its relationship to adjacent structures [3]. Managing ependymomas is challenging, but surgical resection is the most common treatment [5]. This approach aims to remove as much tumor tissue as possible to prevent recurrence and alleviate neurological symptoms [5]. Recurrence occurs in about 50% of cases, typically within 13 to 25 months, and is mainly local, with metastasis in 20% of cases. Therefore, close follow-up is essential to monitor for recurrence [5].

In this case, we present a 29-year-old male with an acute presentation of progressive lower back pain and neurological impairment, diagnosed with MPE via MRI. The patient underwent successful surgical resection, resulting in notable postoperative recovery and symptom relief. This case underscores the critical importance of early diagnosis and intervention in the management of MPE to prevent irreversible neurological damage and improve patient outcomes.

## Case presentation

A 29-year-old male patient presented to the Accident and Emergency Department with progressive lower back pain that started two days prior. The pain was sharp, constant, and radiated to the groin and the anterior and posterior aspects of both thighs. The patient reported that the pain was progressively worsening, and he was unable to bear weight on both legs. He noted that the pain was exacerbated by movement and relieved by lying on his back. He had tried ordinary painkillers but reported that they did not ease the pain. There were no symptoms of urinary or stool incontinence or retention. The patient is normally fit and well, regularly going to the gym every two days. His medical history was unremarkable, with no prior episodes of similar pain or neurological deficits.

Upon examination, the patient had intact sensation in both legs, but motor strength was impaired, scoring 2/5 in both legs. The leg raise test was also impaired bilaterally. Hyperreflexia of the knee reflex was noted bilaterally. A per rectum (PR) examination showed diminished anal tone. Based on a comprehensive neurological assessment and medical history review, there was a strong suspicion of a spinal cord compression pathology. To confirm this diagnosis and exclude differentials like multiple sclerosis, transverse myelitis, Guillain-Barré syndrome (GBS), and B12 deficiency, a spinal MRI was conducted.

Diagnostic assessment

**Figure 1:**
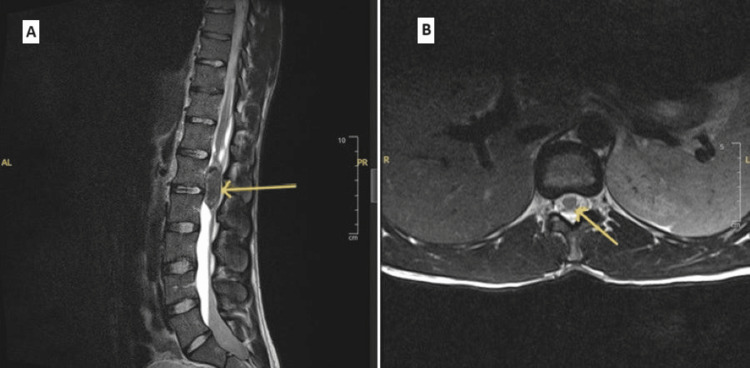
MRI of the spine with IV contrast showing a sagittal view (A) and axial view (B). There is a well-defined, oval-shaped intradural lesion arising from the cauda equina. This lesion extends from the L1 to L2 vertebral level and is most consistent with a myxopapillary ependymoma (denoted by yellow arrows).

The patient's blood test results, as shown in Table 1, were unremarkable. MRI of the whole spinal with contrast showed an intradural lesion, originating from the cauda equina, presenting a clearly delineated oval-shaped lesion with dimensions approximately measuring 4.3 cm craniocaudally (CC), 1.4 cm anteroposteriorly (AP), and 1.5 cm transversely (TR). It extends from the L1 to L2 vertebral level, displaying isointense signals on T1 and heterogeneously hyperintense signals on T2 imaging, with low signal intensity at the tumor margins creating a cap sign (this is highlighted in Figure 1). Following contrast administration, there is minimal enhancement observed in the post-contrast study. The imaging characteristics of the tumor, including its location and appearance, supported the diagnosis of an MPE, necessitating prompt surgical intervention to prevent further neurological deterioration.

**Table 1:** Lab parameters Hb: hemoglobin; WBC: white blood cell count; Cr: creatinine; Na: sodium; K: potassium; CRP: C-reactive protein

Lab Parameters	Value	Normal Range
WBC	16.4	4.5-11 x10^9^/L
Platelets	215	150-400 x10^3^/µL
Urea	4.2	2.1 to 8.5 mmol/L
Cr	102	59-104 μmol/L
Na	144	136 to 145 mmol/L
Hb	162	14 to 18 g/L
K	4.0	3.6-5.2 mmol/L
CRP	8.0	below 5 mg/L

Treatment

**Figure 2:**
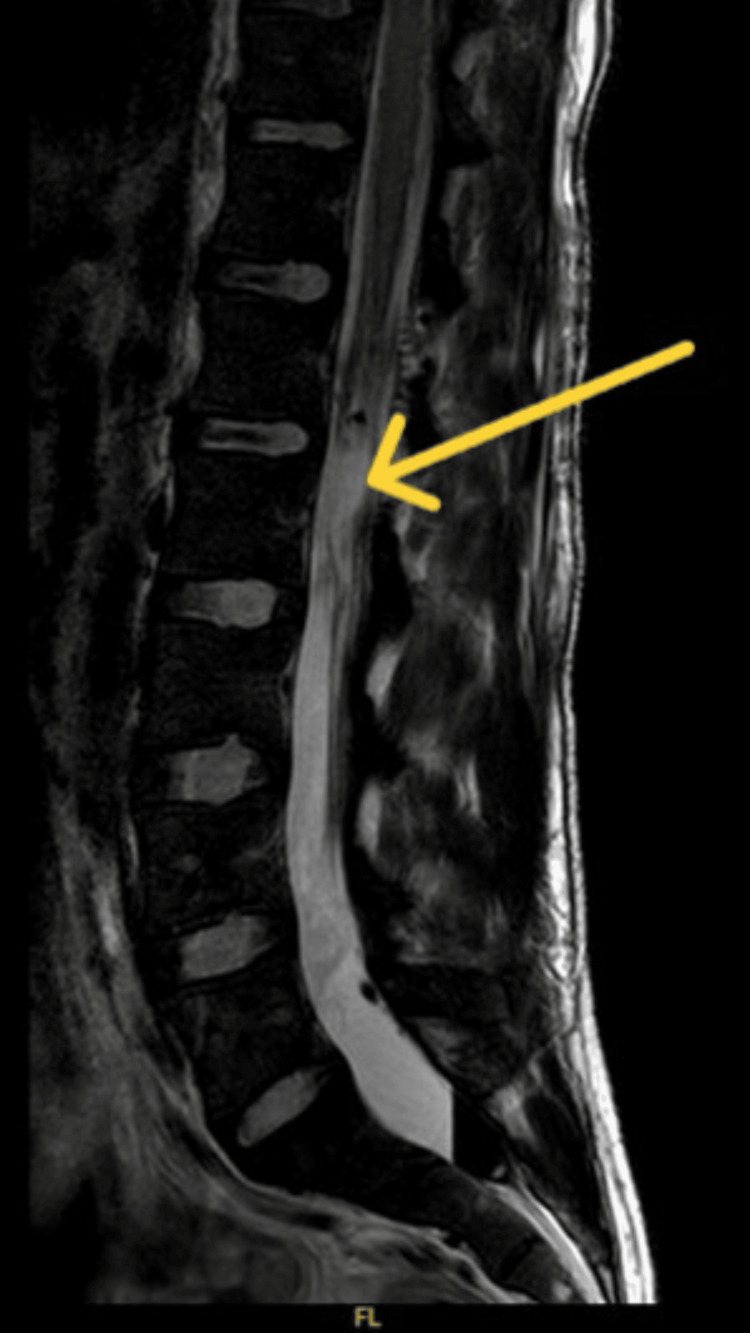
Postoperative imaging demonstrates the resection of the previously noted intradural tumor at the L1/L2 level (denoted by yellow arrow).

The patient underwent a laminectomy and microsurgical resection of the tumor at the level of L1/L2. The primary goal of the surgery was to achieve complete resection of the tumor to relieve spinal cord compression and mitigate the associated symptoms. Intraoperative neuromonitoring was utilized to minimize the risk of neurological injury during the procedure. Gross total resection (GTR) was achieved, confirmed by intraoperative findings and postoperative MRI (Figure 2).

Outcome and follow-up

**Figure 3:**
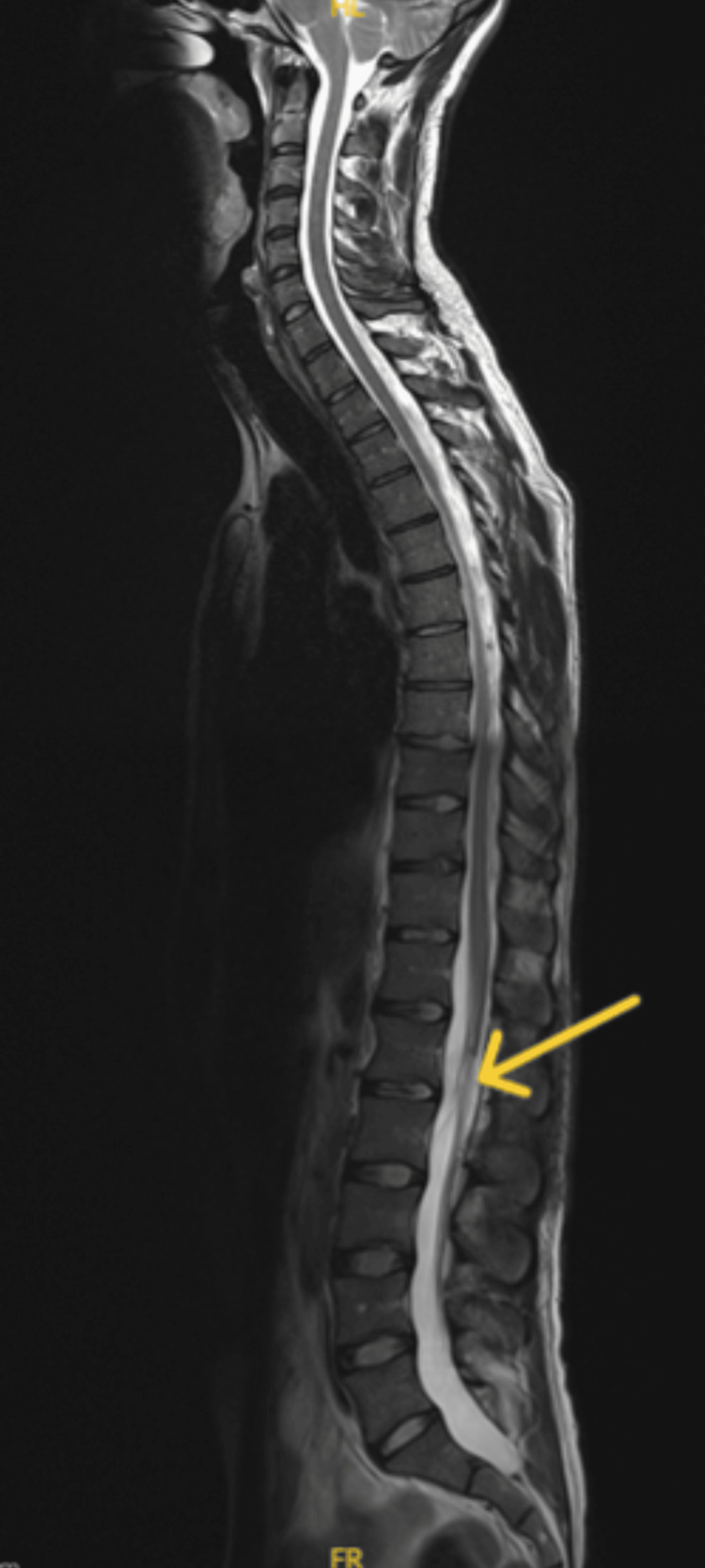
Postoperative imaging demonstrates the resection of the L1/L2 tumor with no convincing evidence of residual tumor (denoted by yellow arrow). Additionally, there is no evidence of disseminated disease.

Postoperatively, microscopic analysis of the specimen showed clusters of abnormal tissue with cells forming elongated structures. The neoplastic cells had a cuboidal to spindle-like shape and were notably arranged around central myxoid regions. Furthermore, immunohistochemical staining was conducted, showing positive results for glial fibrillary acidic protein (GFAP) and localized positivity for epithelial membrane antigen (EMA). These staining patterns support the diagnosis of an intramedullary spinal MPE, classified as grade II. The patient demonstrated gradual improvement in lower limb strength, with motor strength improving to 4/5 over two weeks. There was also a significant reduction in back pain. The patient was mobilized with the assistance of physical therapy, which focused on strengthening and improving mobility. Follow-up MRIs for the whole neuroaxis at one month showed no evidence of residual, recurrent, or metastatic tumor (Figure 3).

## Discussion

A 29-year-old male with a history suggestive of poor prognosis in terms of neurological function, such as progressive lower back pain radiating to the leg and associated motor weakness, hyperreflexia, and diminished anal tone, was diagnosed with an intradural lesion at the cauda equina, which was compatible with an MPE. The patient underwent surgical intervention with a laminectomy and microsurgical resection.

Postoperative recovery featured a dramatic improvement in motor function and alleviation of pain, without evidence of tumor recurrence in subsequent MRIs. MPE is a rare, slow-growing tumor that originates in the lumbosacral region of the spinal cord and more often involves the filum terminale or cauda equina. It is a rare form of spinal cord tumor representing less than 1% and appears to be more common in adults between the ages of 30-50. These tumors are mostly benign in histology, although they may be associated with morbidity because of their location and propensity for local abutment or invasion as well as CSF dissemination [6].

Presentation of MPE is due to mass effect on surrounding organs including lower back pain, radicular signs and symptoms, as well as neurological deficits [7]. In our case, the patient presented with lower back pain, suggesting compression at the L1-L2 nerve root level. The pain radiated to the groin and both thighs, consistent with radicular pain from L1 to L2 nerve root involvement. The patient was unable to bear weight due to significant weakness in both lower limbs, particularly with impaired hip flexion, which is controlled by the L2 segment via the iliopsoas muscle. Despite this, knee extension (L3), ankle dorsiflexion (L4 and L5), lower limb sensation, and bladder function remained intact. However, a reduction in anal tone was noted. These findings strongly point to a compressive lesion at the L1-L2 level. MRI remains the gold standard for evaluating spine cord tumors as it can provide high-contrast and detailed visualization of both the extent of a tumor (including soft-tissue involvement) and its relationship with the spinal cord or other structures [8].

MPE is treated primarily using surgical resection, with GTR being the desired outcome to alleviate symptoms and reduce the risk of recurrence [9]. Intraoperative neuromonitoring is indispensable for providing this protection during surgery [10]. In this scenario, the patient underwent a laminectomy and microsurgical resection with postoperative MRI demonstrating GTR.

Based on the current gold standard, surgical resection is still regarded as preferable for individuals with symptomatic neurological deficits [11]. Nevertheless, patients are recommended to have long-term or life-long follow-up for the purpose of surveillance for any recurrent disease because it can happen despite an apparent complete removal of the tumor [9]. Follow-up investigations, including clinical and MRI assessments, are recommended at one month, three months, six months, and annually to confirm ongoing recovery and re-evaluate for possible recurrence or residual disease [12].

A study on spinal MPE found that approximately one-third of patients experienced treatment failure. Recurrence patterns were mainly local, but many patients also experienced nonlocal failure. Younger patients and those not initially treated with adjuvant radiotherapy or not undergoing GTR were more likely to experience tumor recurrence or progression. The estimated 10-year overall survival was 92.4%, and the 10-year progression-free survival was 61.2%. Treatment modality and extent of surgery were identified as prognostic factors for local control and progression-free survival [13].

Adjuvant radiation therapy for spinal MPEs appears to play a critical role in improving treatment outcomes. A study suggests that radiation therapy may be valuable as salvage therapy, helping to delay recurrences of spinal MPEs [14]. Another study found that postoperative high-dose radiation therapy significantly reduces local recurrence rates and the risk of concomitant spinal or brain metastasis in patients with primary spinal MPEs, highlighting its effectiveness in improving overall treatment efficacy [15].

## Conclusions

Diagnosing MPE presents significant challenges, emphasizing the need for early detection and effective management. Spinal tumors including MPE should be considered in patients presenting with severe lower back pain, motor weakness, hyperreflexia, and reduced anal tone. Prompt MRI is critical for accurate diagnosis and assessment of tumor extent, and it should not be delayed. Early surgical intervention is essential for achieving curative outcomes, alleviating symptoms, reducing recurrence risk, and improving overall prognosis. Lifelong follow-up is mandatory to monitor for early signs of recurrence and ensure sustained recovery. Moreover, ongoing research into adjuvant therapies, such as radiation, continues to evolve treatment strategies and enhance outcomes for patients with this complex spinal tumor.
